# Impaired Limb Functional Outcome of Peripheral Nerve Regeneration Is Marked by Incomplete Recovery of Paw Muscle Atrophy and Brain Functional Connectivity in a Rat Forearm Nerve Repair Model

**DOI:** 10.1155/2021/6689476

**Published:** 2021-02-11

**Authors:** Qiyuan Bao, Qi Liu, Jun Wang, Yuhui Shen, Weibin Zhang

**Affiliations:** ^1^Department of Orthopaedics, Shanghai Ruijin Hospital, Shanghai Jiao Tong University School of Medicine, Shanghai, China; ^2^Department of Orthopaedics, Shanghai Institute of Traumatology and Orthopaedics, Shanghai, China

## Abstract

Skilled sensorimotor deficit is an unsolved problem of peripheral nerve injury (PNI) led by limb trauma or malignancies, despite the improvements in surgical techniques of peripheral nerve anastomosis. It is now accepted that successful functional recovery of PNI relies tremendously on the multilevel neural plasticity from the muscle to the brain. However, animal models that recapitulate these processes are still lacking. In this report, we developed a rat model of PNI to longitudinally assess peripheral muscle reinnervation and brain functional reorganization using noninvasive imaging technology. Based on such model, we compared the longitudinal changes of the rat forepaw intrinsic muscle volume and the seed-based functional connectivity of the sensorimotor cortex after nerve repair. We found that the improvement of skilled limb function and the recovery of paw intrinsic muscle following nerve regeneration are incomplete, which correlated with the functional connectivity between the primary motor cortex and dorsal striatum. Our results were highly relevant to the clinical observations and provided a framework for future investigations that aim to study the peripheral central sensorimotor circuitry underlying skilled limb function recovery after PNI.

## 1. Introduction

Skilled sensorimotor deficit is a common yet unsolved clinical problem of peripheral nerve injury (PNI) [1–4] and surgical repair (PNR) [1, 5, 6] due to limb trauma or extremity malignancy. Despite improvements in surgical techniques to foster peripheral nerve regeneration, PNI still leads to long-lasting disabilities in terms of lost fine sensorimotor function [6, 7]. Previous human studies suggest that the brain undergoes a sensorimotor relearning process after peripheral nerve regeneration, and such misorganization and reorganization process could significantly impact the outcome of nerve regeneration [8, 9]. It is now increasingly accepted that successful peripheral nerve rehabilitation is, rather than simply a matter of surgical reconstruction, a combinatorial effort of the integrated treatment strategies targeting the plasticity from the muscle to the brain [10, 11]. However, the animal models that recapitulate these processes are still lacking. To date, the mechanistic details of the coordination and reorganization of the peripheral reinnervation and central plasticity after PNI are still poorly understood.

In this report, we developed a rat model of PNI to assess the peripheral muscle reinnervation and brain functional reorganization process longitudinally using noninvasive imaging technology, with the skilled forelimb function recovery monitored using ladder rung walking task [12]. Using such model, we analyzed the longitudinal changes of the rat forepaw intrinsic muscle and the functional connectivity of the primary motor cortex after PNI and PNR, to interrogate the potential neural mechanism of the sensorimotor circuitry underlying the limb functional recovery.

## 2. Methods

### 2.1. Animals

For both limb muscle reinnervation model and the paw intrinsic muscle reinnervation model, 15 adult male Sprague-Dawley rats weighing 200–250 grams were randomly assigned to the control (*n* = 5), nerve injury (*n* = 5), and nerve repair (*n* = 5) groups. Experiment animals were given access to food and water ad libitum and housed with soft bedding on a 12 h light/dark cycle in a temperature-controlled environment (21 ± 2°C). Rats were handled regularly before any surgical procedures, MR imaging, and behavioral testing, and were sacrificed at week 10 following the behavioral assessment.

### 2.2. Limb Muscle Reinnervation Model

**Figure 1:**
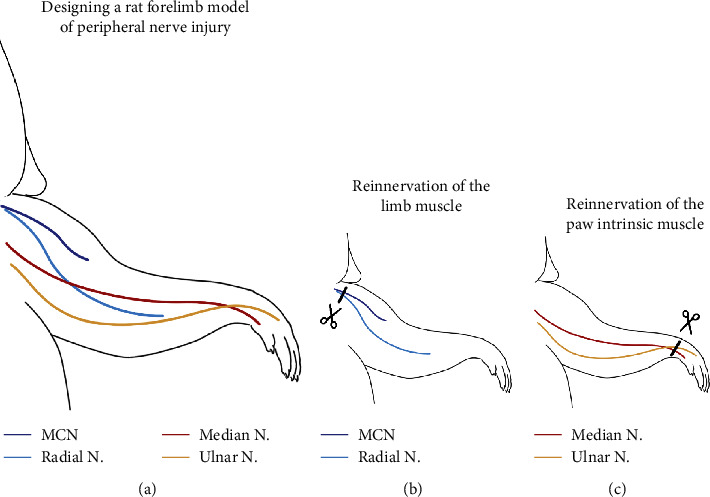
The surgical procedure of the raw forepaw. (a) To assess the skilled functional recovery outcome after peripheral neural repair, we designed a nerve anastomosis model using rat forelimb. (b) The musculocutaneous nerve (MCN) and radial nerve of the rat forelimb were resected with or without nerve repair, to investigate the impact of limb muscle denervation and reinnervation on the skilled limb function. (c) Similarly, the median nerve and the ulnar nerve were manipulated to study the denervation and reinnervation of the paw intrinsic muscle.

To investigate the skilled functional outcome, limb muscle reinnervation model was designed (Figures 1(a) and 1(b)). Briefly, all rats were anesthetized with isoflurane (1.5–2%) and a mixture of 30% N_2_O and 70% O_2_. With the longitudinal incision at the right proximal upper limb, the musculocutaneous nerve (MCN) and the radial nerve were exposed before their innervation of the biceps muscle and the triceps muscle, respectively. For the nerve injury group, both nerves were transected and tightly ligated to prevent the nerve stump regeneration. For the nerve repair group, both nerves were exposed in the same fashion. Then, the stumps of both nerves were immediately anastomosed through a 10-0 monofilament epineurial microstitch (Ethicon, USA) following the nerve transection. For the control group, sham operations were performed, where MCN and radial nerve were exposed as in the injury group, but received no further manipulations.

### 2.3. Paw Intrinsic Muscle Model

The paw intrinsic muscle reinnervation model was established to investigate its impact on the skilled functional outcome after PNI (Figures 1(a) and 1(c)). Under anesthesia, a longitudinal incision was placed at the right distal forearm of the rat. The medial and ulnar nerves were explored at the wrist level. Both of the nerves were transected and ligated in the nerve injury group, or transected and then repaired through a 10-0 monofilament epineurial microstitch (Ethicon, USA) in the repair group. In contrast, the nerves were only exposed without any further procedure in the control (sham) group.

### 2.4. Ladder Rung Skilled Walking Task

Prior to the behavioral testing, rats were handled and trained to acclimate the ladder for 7 consecutive days in the ladder rung apparatus, which was made according to the original report [13]. The apparatus was composed of self-made horizontal ladder rungs between the transparent sidewalls. 51 ladder rungs in 2 cm intervals (a total length of 1 m) were presented to the rat toward the home cage. During the walking of the rats, we performed video recording for each walking trial of the animal to capture the foot errors, including slight slip, deep slip, and total miss of the step. Skilled limb sensorimotor function was measured as the number of errors divided by the number of total steps for the affected limb, averaged for five consecutive trials. The foot fault rate between groups was compared using Student's *t*-test, with a *p* value < 0.05 considered statistically significant.

### 2.5. MR Scanning Parameters of the Rat Brain

MR imaging of the rat forepaw and brain was carried out with a Bruker 7 T/40 cm horizontal magnet (ClinScan, Bruker BioSpin, Germany). The functional changes of the rat brain were studied using parameters as previously reported [14]. Briefly, anesthesia was given to the rats during the experiments with isoflurane (1.75–2.5%) mixed with air. The respiratory rate and heart rate were monitored and recorded during scans. The rat body temperature was maintained and surveilled at about 37°C during the imaging session. MRI scans were started when physiological parameters stabilized for at least 10 minutes. Blood oxygen level-dependent (BOLD) contrast-sensitive T2∗-weighted gradient-echo echo-planar images were acquired for resting-state fMRI scans. Each scan consisted of 300 volumes of 14 slice acquisitions (repetition time (TR) of 1.3 seconds, echo time (TE) of 25 milliseconds, flip angle = 60°, 1.0 mm slice thickness, and 0.5 × 0.5 mm^2^ in-plane resolution). A high-resolution T2-weighted RARE anatomical reference was acquired for each animal (1.0 mm slice thickness and 0.273 × 0.273 mm^2^ in-plane resolution). An additional T2-weighted RARE anatomical scan with the same geometry as the functional image (1 mm slice thickness and 0.5 × 0.5 mm^2^ in-plane resolution) was also acquired and used as a low-resolution anatomical reference.

### 2.6. MR Scanning Parameters of the Rat Forepaw

After the MRI scan of the rat brain, imaging of the rat forepaw was immediately conducted. A total of four sequencing protocols were tested and compared in the preliminary study: (1) the turbo spin echo (TSE) sequence (parameter 1) with an in-plane resolution of 0.27 × 0.27 mm^2^, (2) gradient echo (GRE) sequence with an in-plane resolution of 0.99 × 0.5 mm^2^, (3) GRE sequence with an in-plane resolution of 0.5 × 0.5 mm^2^, and (4) GRE sequence with an in-plane resolution of 0.15 × 0.15 mm^2^ (Table 1). MR images of the rat forepaw were given to four independent readers, who were asked to report their confidence in the manual segmentation of the intrinsic muscle with a ranking of 1 (worst) to 4 (best) assigned to the four scanning parameters. The scanning parameter with the highest ranking was chosen for the formal study, where rat brain functional imaging and rat forepaw muscle volumetry imaging were acquired for each of the rats.

### 2.7. Image Preprocessing

Resting-state fMRI data were preprocessed with FSL 5.1 (https://www.fmrib.ox.ac.uk/fsl) [15]. The fMRI was skull stripped and preprocessed with signal despiking, removal of physiological artifacts from respiration and heartbeat, and correction for slice timing. Preprocessed images were then corrected for motion, followed by spatially smoothing with a Gaussian kernel of 0.8 mm FWHM and high-pass filtering with a cutoff of 100 seconds. Volumes from functional images were registered to a standard space with a three-step process. Images were first aligned with the individual's low-resolution anatomical image, followed by alignment with the individual's high-resolution anatomical image, and then coregistered to a standard space. Average time courses from all of the voxels inside white matter (WM) and cerebrospinal fluid (CSF) were extracted. Confounding regressors that modeled WM and CSF signals and six motion parameters, including translations and rotations, were removed from the images through linear regression.

### 2.8. Seed-Based Resting-State Functional Connectivity (FC) Analysis

Whole-brain resting-state connectivity of the region of interest (ROI) was constructed as described in [14, 16]. Briefly, a standard rat atlas [17] comprising 96 anatomical ROIs (48 regions in each hemisphere) was used as the reference. The contralateral (left) primary motor cortex was chosen as the seed for analyzing the whole-brain FC. We then extracted the time course of BOLD signal across all voxels within a given ROI and generated the mean time course for each ROI. The functional correlation was computed using the Pearson correlation coefficient between the time courses of the seed with every other ROI. Correlation coefficients were transformed to **z**-scores using Fisher's **z** transformation as the final FC value for each subject. The FC strength was compared between the corresponding connections using FSL FEAT analysis to find significant brain regions. The FC with a **p** value < 0.05 after multiple comparison correction was considered statistically significant.

## 3. Results

### 3.1. The Recovery of Skilled Forelimb Function following Muscle Denervation and Reinnervation

**Figure 2:**
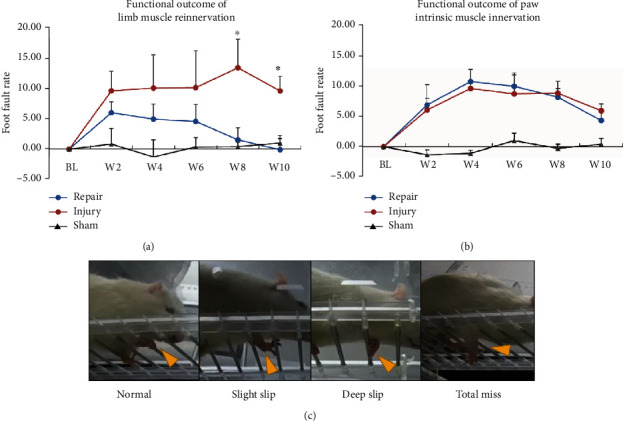
The functional outcome of the skilled limb movement after peripheral nerve injury. (a) In the limb muscle reinnervation design, the foot fault rate of the nerve repair group was observed to be indifferent from the sham group at W8 and W10, but significantly lower than that of the injury group. (b) In the intrinsic muscle reinnervation design, we found no recovery of the skilled foot function in the repair group compared to the injury group, resulting in a significantly higher foot fault rate than the sham group. (c) Exemplary patterns of various degrees of foot fault (yellow arrow) in rat forelimb during skill walking. ^∗^*p* < 0.050. ns: nonsignificant.

We first compared the effect of limb muscle (large muscle) reinnervation versus paw intrinsic muscle (small muscle) reinnervation on the functional recovery of the skilled forelimb performance. In both large muscle model and small muscle model, the nerve injury group exhibited an increased foot fault rate compared to the baseline (*p* < 0.05) postsurgery. In contrast, the skilled forelimb functional recovery demonstrated a highly different pattern between the two model designs. In the large muscle model, we observed a gradual recovery of the foot fault rate in the repair group after surgery. The foot fault rate of the repair group was significantly lower than that of the injury group at week 8 (*p* = 0.049) and week 10 (*p* = 0.038) (Figure 2(a)). However, the similar trend was not seen in the repair group of the small muscle model. Various degrees of impaired forepaw positioning (slight and deep slip) and compensatory adjustment ability (total miss) were observed (Figure 2(c)). The foot fault rates of the injury and the repair groups were not significantly different postoperatively from week 2 to week 10 (*p* > 0.05) (Figure 2(b)), suggesting a defective functional recovery of the intrinsic muscle compared to the large muscle following PNI.

### 3.2. Establishing the MR Imaging Paradigm for Noninvasive Assessment of the Paw Intrinsic Muscle Volume

**Figure 3:**
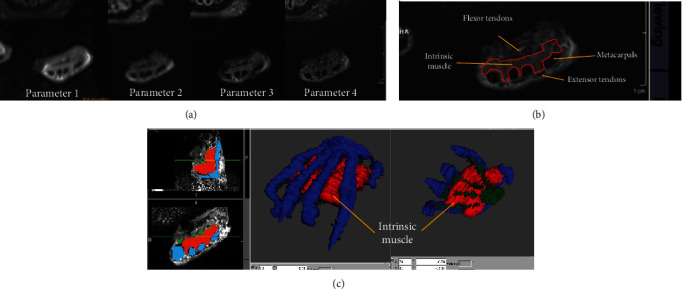
Establishing the MR imaging paradigm to assess the paw intrinsic muscle volume. (a) To determine the best imaging protocol, we compare the imaging quality of the four parameters specified in Table 1. Readers were asked to report their confidence in the manual segmentation of the intrinsic muscle, which was contoured by the metacarpal bone and the flexor/extensor tendons (b). (c) Finally, manual segmentation of the intrinsic muscle (red voxels) based on parameter 4 was taken as the final setup for muscle volumetry of the subsequent study.

Since there were no established MR parameters for imaging rat paw intrinsic muscle, we prioritized the optimal sequencing parameters from a total of four potential settings (Table 1 and Figure 3(a)) based on the imaging quality and time consumption. Due to the fact that rat forepaw intrinsic muscle is contoured by the metacarpal bone and the flexor/extensor tendons (Figure 3(b)), we then asked four independent readers to report their confidence in the manual segmentation of the intrinsic muscle contour based on the MR imaging with the aforementioned four parameters. The turbo spin echo (TSE) sequence (parameter 1) achieved an acceptable imaging quality, yet at the expense of an extremely prolonged acquisition time (>28 min). Therefore, further optimization based on TSE sequence was not attempted. Then, gradient echo (GRE) sequence was performed with three sets of parameters, at the resolution of 0.99 mm × 0.5 mm, 0.5 mm × 0.5 mm, and 0.15 mm × 0.15 mm, respectively. Interestingly, all four readers assigned parameter 4 as the highest quality one (parameter 4 versus the other, *p* = 0.025) (Table 1). Therefore, we chose parameter 4 with manual segmentation of the muscle contour (Figure 3(c)) as the final setup for volumetry of paw intrinsic muscle in the subsequent study.

### 3.3. Brain Functional Reorganization Analysis of the Primary Motor Cortex (M1)

**Figure 4:**
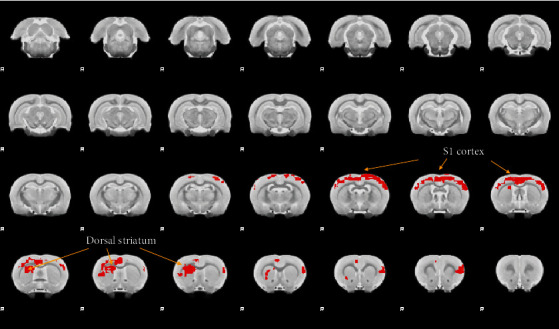
Brain functional reorganization analysis of the primary motor cortex (M1) after nerve repair. We performed a seed-based whole-brain functional connectivity (FC) analysis of the contralateral primary motor cortex (M1) between the nerve repair and the nerve injury groups (repair-injury). The rat contralateral M1 cortex (left) demonstrated an extensive FC reorganization (marked in red) with bilateral sensory cortex (S1) as well as right dorsal striatum (dS) at week 8.

To investigate the brain functional reorganization process following peripheral reinnervation of the paw intrinsic muscle, we performed a seed-based whole-brain functional connectivity (FC) analysis of the contralateral primary motor cortex (M1) between the nerve injury and the nerve repair groups. There was no significant difference of connections involving the M1 cortex at week 6. Interestingly, the rat contralateral M1 cortex (left) demonstrated an extensive FC reorganization with the left sensory cortex (S1), the right sensorimotor cortex (S1M1), and the right dorsal striatum (dS) (Figure 4) at week 8. Remarkably, the dorsal striatum has been reported as a principal structure facilitating the neural plasticity underlying the locomotion function [18–20], supporting our hypothesis that dS-M1 connectivity change might be a critical central reorganization process of peripheral reinnervation following PNI.

### 3.4. Tracing the Dynamics of dS-M1 Connectivity and Paw Reinnervation following PNI

**Figure 5:**
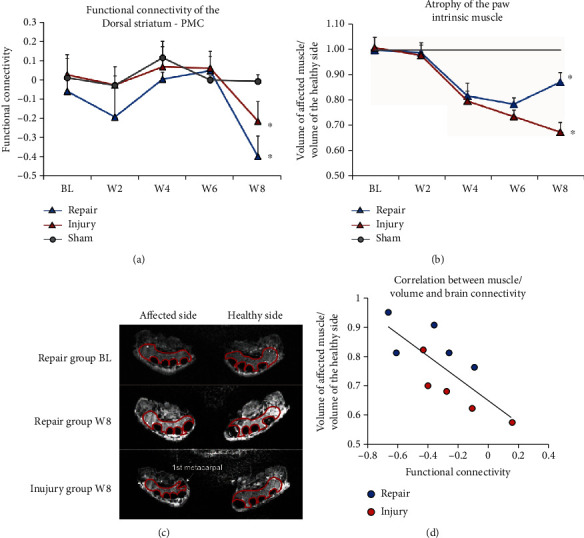
The change of dS-M1 connectivity and paw muscle atrophy following PNR. (a) The FC of the dS-M1 remains similar among the three groups until week 8, when the FC of the nerve injury and the repair group was statistically different from that of the sham group. (b) The degree of muscle atrophy was gradually aggravated from week 2 postsurgery in the nerve injury and repair groups. However, the nerve repair group demonstrated only partial recovery of muscle volume at week 8. (c) The muscle atrophy of the repair and the injury groups was shown in an exemplary graph. Note that remarkable muscle atrophy of the injured side was observed at week 8 in the injury group, but not in the repair group. The adduction of the 1st metacarpal was also observed due to the thenar atrophy of the paw. (d) We observed a significant correlation between the dS-M1 FC and the degree of muscle atrophy in rats with nerve injury and repair at week 8. ^∗^*p* < 0.05 compared to the control.

We then asked whether the dS-M1 functional reorganization was in parallel with peripheral reinnervation of the rat forelimb intrinsic muscle. The degree of muscle atrophy was gradually aggravated from week 2 postsurgery. However, the muscle atrophy of the nerve repair group demonstrated a partial recovery at week 8 (Figure 5(b)), which was significantly higher than that of the nerve injury group (*p* = 0.020). In contrast, the atrophy of the affected intrinsic muscle in the injury group was further exacerbated at week 8 (Figure 5(c)). Accordantly, we observed a highly similar trend of dS-M1 plasticity dynamics. The FC change of the nerve repair group was indifferent from either the nerve repair or the sham group before week 6. However, dS-M1 FC of the repair group and the injury group was statistically different from that of the sham group at week 8 (*p* < 0.05) (Figure 5(a)). Interestingly, we observed a significant correlation between the dS-M1 FC and the degree of muscle atrophy (*r* = 0.601, *p* = 0.011) (Figure 5(d)), suggesting potential neural feedback and functional circuitry of the peripheral reinnervation with central plasticity.

## 4. Discussion

Numerous animal models have been established in the previous literature for assessing the loss and recovery of limb skilled locomotion function after brain [21] or spinal cord injury [22]. However, rodent studies regarding central plasticity following PNR are surprisingly lacking, which is in sharp contrast with the unmet need for surgical repair and rehabilitative interventions for PNI [23]. In the last decades, a plethora of emerging treatment modalities that target central plasticity has been investigated, such as activity-based sensory reeducation, selective deafferentation, crossmodal sensory substitution, and mental motor imagery [23, 24]. While the importance of strengthening the central relearning process during the sensorimotor reinnervation and skilled function recovery has been increasingly recognized, novel animal models were needed to unravel these mechanisms. Our study, for the first time, proposed a paradigm using noninvasive MR imaging to monitor the peripheral reinnervation and central functional reorganization in a longitudinal manner. Our finding also demonstrated that the ladder rung walking task, which was originally designed for brain or spinal cord injury measurement [12], can be implemented to faithfully capture the skilled limb function following PNI and regeneration.

Unlike previous models of sciatic nerve regeneration, our findings highlighted the incomplete recovery of the intrinsic muscle atrophy and skilled sensorimotor deficit. Interestingly, reinnervation of the large limb muscle caused by the MCN and radial nerve repair also failed to capture such suboptimal neurorehabilitation outcomes. Hypothetically, this discrepancy is likely due to a higher degree of loss or misdirected sensory input following axonal regrowth, resulting in a heavier mismatch of the cortical representation of the reinnervated forepaw [24]. Furthermore, the cortical representation of the paw sensorimotor function was much larger than that of the limb, which enables a greater encroachment of the adjacent intact cortical areas into the nerve-injured cortex [25]. It is reported that such extensive maladaptive plasticity has a limited role in and might even jeopardize the process of the sensorimotor function outcome [24]. Additionally, our model emphasized on skilled locomotion, rather than gross muscle function following PNR. Such a recovery process demands more complex and extensive central plasticity in the nervous system, which is reinforced by our findings that the FC changes of M1 with dS and S1M1 regions occur at a later stage and correlate with the temporal dynamics of muscle atrophy recovery. Finally, the denervation atrophy was hypothesized to be more irreversible for the paw intrinsic muscle, compared to that for the large muscle [26] after neural regeneration.

To our knowledge, this is the first report examining the dynamics of paw intrinsic muscle reinnervation and central reorganization in parallel using the rat peripheral nerve model. Our results were highly relevant to clinical observations of the suboptimal outcome of skilled hand functional recovery [27, 28]. Further studies are warranted at the cellular and neural circuit levels to determine the peripheral central feedback mechanisms, as well as the potential therapeutic targets underlying the functional rehabilitation after peripheral nerve reinnervation.

## Figures and Tables

**Table 1:** Establishing the optimal sequencing parameter for measuring the forepaw intrinsic muscle volume.

Parameter	Sequence	Resolution (mm)	FoV read (mm)	FoV phase (%)	Slice thickness (mm)	SNR	TR (ms)	TE (ms)	Confidence of the segmentation					Total time (mm:ss)
									Rater 1	Rater 2	Rater 3	Rater 4	Average score	
1	TSE^∗^	0.27 × 0.27	35.00	79.70	1.20	1.00	2000	24	1	2	2	1	1.5	28:18
2	GRE	0.99 × 0.50	130.00	100.00	1.00	5.25	28	4	2	1	1	2	1.5	13:18
3	GRE	0.50 × 0.50	129.00	40.00	0.50	4.64	28	4	3	3	3	3	3.0	11:35
4	GRE	0.15 × 0.15	40.00	53.10	0.15	1.22	28	4	4	4	4	4	4.0	17:23

^∗^We did not further increase the resolution of the TSE scanning sequence, due to the tremendous amount of imaging time (>28 min) at this parameter.

## Data Availability

The data used to support the findings of this study are available from the corresponding author upon request.
